# Age-stratified management of pediatric perianal abscesses: a proposed framework integrating the “Developmental Healing Axis”

**DOI:** 10.3389/fped.2026.1789225

**Published:** 2026-05-15

**Authors:** He-Xue Yuan, Zhi-Tao Yin, Li-Hua Wang, Lin Ma, Yang Liu, Yu-Lin Ju, Bin Yue

**Affiliations:** Department of Anorectal Surgery, Shenyang Coloproctology Hospital, Shenyang, Liaoning, China

**Keywords:** age-specific management, fistula-in-ano, incision and drainage (I&D), minimally invasive surgery, non-cutting seton, pediatric perianal abscess, spontaneous resolution

## Abstract

Pediatric perianal abscesses represent a frequent yet underrecognized condition that poses diagnostic and therapeutic challenges due to tissue fragility and age-dependent healing dynamics. This review synthesizes current evidence on the epidemiology, pathophysiology, and management of pediatric perianal abscesses to provide a conceptual basis for clinical decision-making. Surgical interventions such as incision and drainage offer rapid symptom relief but may increase the risk of fistula formation, particularly in older children. In contrast, conservative strategies including antibiotic therapy and observation are often effective in infants and young children, reflecting their superior regenerative capacity. Age-related differences in immune response, microbiota composition, and glandular anatomy contribute to variations in disease progression and healing outcomes. By integrating data from recent studies and clinical guidelines, this review highlights the importance of individualized, age-specific management to optimize recovery, minimize recurrence, and prevent fistula development. Furthermore, it identifies gaps in current evidence and underscores the need for standardized treatment protocols and future research into predictive and microbiome-related factors influencing disease course.

## Highlights

1. Infants benefit from a self-healing capacity driven by immune immaturity and regenerative predominance; conservative management (CM) is therefore preferred.
2. Incision and drainage (I&D) offer rapid symptom relief but carries a moderate risk of recurrence.
3. Incision, drainage, and fistulotomy (IDF) achieves high cure rates, yet may represent overtreatment for transient disease in infants.

## 1 Introduction

Perianal abscesses, defined as localized collections of pus in the perianal region, are clinically important conditions affecting both adults and children (1–3). In pediatric populations, these abscesses present unique challenges due to delicate anatomy, heightened tissue sensitivity, and age-dependent healing responses. Epidemiological studies estimate an incidence of 0.5–3% in infants, with the highest occurrence in children under five years of age and a male-to-female ratio of approximately 2:1 (4, 5). Certain underlying conditions, including immunodeficiencies and gastrointestinal disorders, further increase susceptibility to infection (6).

The pathogenesis of perianal abscesses typically involves infection of the anal glands, resulting in localized swelling, erythema, tenderness, and pain around the anus (7). Although more common in adults, perianal abscesses are also prevalent in children, where evaluation must include a detailed history and physical examination to identify contributing factors such as systemic illness or immune dysfunction (8). In infants, many cases may resolve spontaneously due to their strong regenerative potential; however, untreated abscesses can progress to complications including fistula-in-ano, cellulitis, or systemic infection, underscoring the importance of early diagnosis and individualized management.

Traditionally, surgical incision and drainage (I&D) have been the cornerstone of treatment, providing rapid symptom relief and reducing the risk of recurrence or progression (9, 10). Nevertheless, this intervention may be associated with postoperative complications, including scarring and fistula formation, particularly in older children. These risks have prompted a shift toward more conservative approaches in younger patients, who exhibit greater tissue regeneration capacity supported by abundant type III collagen. Consequently, non-surgical management, such as antibiotic therapy and conservative care, has gained increasing attention in infants and toddlers. Antibiotics targeting *Staphylococcus aureus* and *Streptococcus* species can effectively treat acute infections, though their long-term role in preventing recurrence remains controversial.

Recent advances in pediatric abscess management emphasize precision and minimally invasive strategies. Innovations such as ultrasound-guided drainage have improved procedural accuracy and minimized damage to surrounding tissues (11, 12). Furthermore, updated clinical guidelines now advocate age-specific therapeutic algorithms that integrate infection control, pain management, and recurrence prevention within a multidisciplinary framework (13–15).

Given the variability in healing capacity, abscess morphology, and underlying etiology among pediatric age groups, individualized treatment remains essential. Optimal management should consider patient age, abscess size, symptom duration, immune status, and recurrence history to balance efficacy and safety across treatment modalities.

This review aims to synthesize current evidence and recent advances in the management of pediatric perianal abscesses, encompassing both surgical and non-surgical strategies. By integrating epidemiological insights, pathophysiological mechanisms, and treatment outcomes, it provides evidence-based guidance for clinicians to optimize therapeutic decisions, improve prognosis, and reduce recurrence and fistula formation in children.

## 2 Literature search strategy

We searched PubMed, Web of Science, and Scopus for articles published over the last 20 years using keywords: “pediatric perianal abscess”, “fistula-in-ano”, and “age-specific management”. Inclusion focused on clinical outcomes and developmental biology studies.

## 3 The “Developmental Healing Axis” as a conceptual framework

These age-dependent distinctions in anatomy, collagen dynamics, and immune regulation are synthesized here into a proposed “Developmental Healing Axis”. This conceptual framework is intended to organize existing clinical observations and provide a structured perspective for future research into pediatric-specific management strategies.

### 3.1 Anatomical and physiological distinctions

Pediatric perianal regions differ fundamentally from adults, extending beyond size to include immature anal glands, shorter anal canals, thinner sphincter muscles, reduced perianal fat, and underdeveloped vascular and lymphatic networks. These features increase susceptibility to blockage and abscess formation, while also heightening sensitivity during surgical procedures. Moreover, congenital fistulas and developmental anomalies are common precursors in children, contrasting with Crohn's disease or trauma in adults.

### 3.2 Immune maturity, growth factor regulation, and microbiome alterations

The pediatric immune system, though immature, demonstrates accelerated yet tightly regulated responses. Collagen abundance enhances both inflammatory and proliferative phases, while growth factors such as TGF-β (16, 17) and PDGF (18) actively drive fibroblast activation, angiogenesis, and extracellular matrix deposition. These pathways foster efficient healing and minimize chronic inflammation (19, 20).

Recent multi-omics evidence further highlights the role of gut microbiota and metabolites in the pathogenesis of pediatric perianal abscesses. A comprehensive study integrating 16S rRNA sequencing and untargeted metabolomics revealed significant differences in microbial diversity and composition between children with PA and healthy controls. Specifically, Enterococcus was elevated, while beneficial genera such as *Faecalibacterium*, *Blautia*, *Fusicatenibacter*, and *Eubacterium hallii group* were depleted. Metabolomic profiling identified 1,168 differential metabolites, with down-regulation of amino acid biosynthesis pathways (phenylalanine, tyrosine, tryptophan; valine, leucine, isoleucine; pantothenate and CoA biosynthesis) and up-regulation of ubiquinone, terpenoid-quinone, tyrosine, and tryptophan metabolism. Correlation analyses confirmed meaningful associations between altered microbial composition and specific metabolic profiles, underscoring the complex interplay between microbiota and host metabolism in pediatric PA (21).

Together, these findings suggest that age-dependent immune regulation is not only shaped by collagen and growth factor signaling but also by characteristic alterations in gut microbiota and metabolites. This integrated perspective provides a foundation for future microbiome-targeted therapeutic strategies in pediatric populations.

## 4 Surgical management strategies: an age-stratified and risk-adapted approach

Surgical management of pediatric perianal abscesses has evolved from a uniform aggressive approach to a nuanced, age-stratified strategy. The primary therapeutic goal is to balance effective drainage and fistula eradication against the imperative of preserving anal sphincter integrity and preventing iatrogenic injury (Table 1).

**Table 1:** Comparison of management strategies for pediatric perianal abscess and fistula.

Parameter	Conservative (CM)	Incision & drainage (I&D)	Incision + fistulotomy (IDF)
Procedure	Observation, hygiene, selective antibiotics	Abscess evacuation without tract exploration	Combined drainage and excision of fistula tract
Primary Indication	Stable infants (<12 m); localized, non-fluctuant lesions	Large abscesses or failure of initial conservative trial	Recurrent cases or clinically confirmed fistula
Rationale	Harnesses age-related healing & immune immaturity	Immediate infection control and decompression	Definitive management of the underlying tract
Typical Age	Infants (<12 m)	Young children (1–6 yrs)	Older children & adolescents
Success Rate	∼80%–95%	∼70%–85%	∼90%–98%
Recurrence	10%–25% (lower in early infancy)	15%–30%	2%–5%
Advantages	Non-invasive; avoids anesthesia risks	Rapid symptom relief; technically simple	Lowest recurrence; definitive cure
Limitations	Requires close monitoring; slow resolution	High recurrence; ignores potential fistula	Invasive; potential risk to sphincter integrity
Recovery	Outpatient; rapid	Short inpatient stay (1–2 days)	Inpatient care; prolonged healing time

### 4.1 Incision and drainage (I&D): the first-line standard

**Figure 1:**
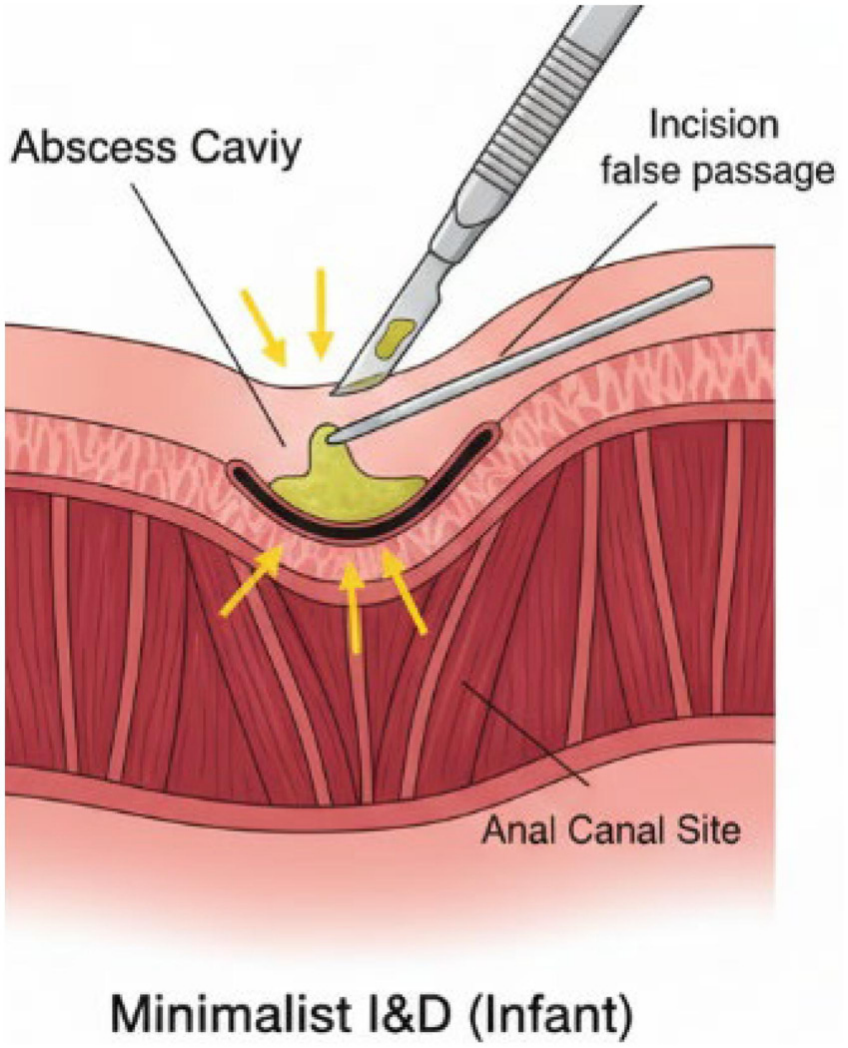
Minimalist incision and drainage (I&D) strategies for pediatric perianal abscesses.

Incision and drainage (I&D) are the most common procedure, providing rapid symptom relief through pus evacuation and tissue decompression (11, 22). However, technical execution varies significantly by age group:

In Infants (<12 months): Given the high prevalence of self-limiting cryptoglandular infections driven by transient androgen surges, current consensus favors a “minimalist incision and drainage (I&D)” strategy (Figure 1). This approach emphasizes a small incision solely for pus evacuation, avoiding aggressive probing or fistula exploration during the acute phase. Emerging evidence from large retrospective cohorts with long-term follow-up indicates that conservative management or limited I&D achieves cure rates comparable to more aggressive surgical interventions, while maintaining a low risk of complications. In contrast, aggressive probing to identify a fistula in the acute setting may significantly increase the risk of creating false tracts and subsequent chronic fistulas, without conferring additional benefit in reducing long-term recurrence rates, thereby representing potential overtreatment in this developmentally self-limiting population (23).

**Figure 2:**
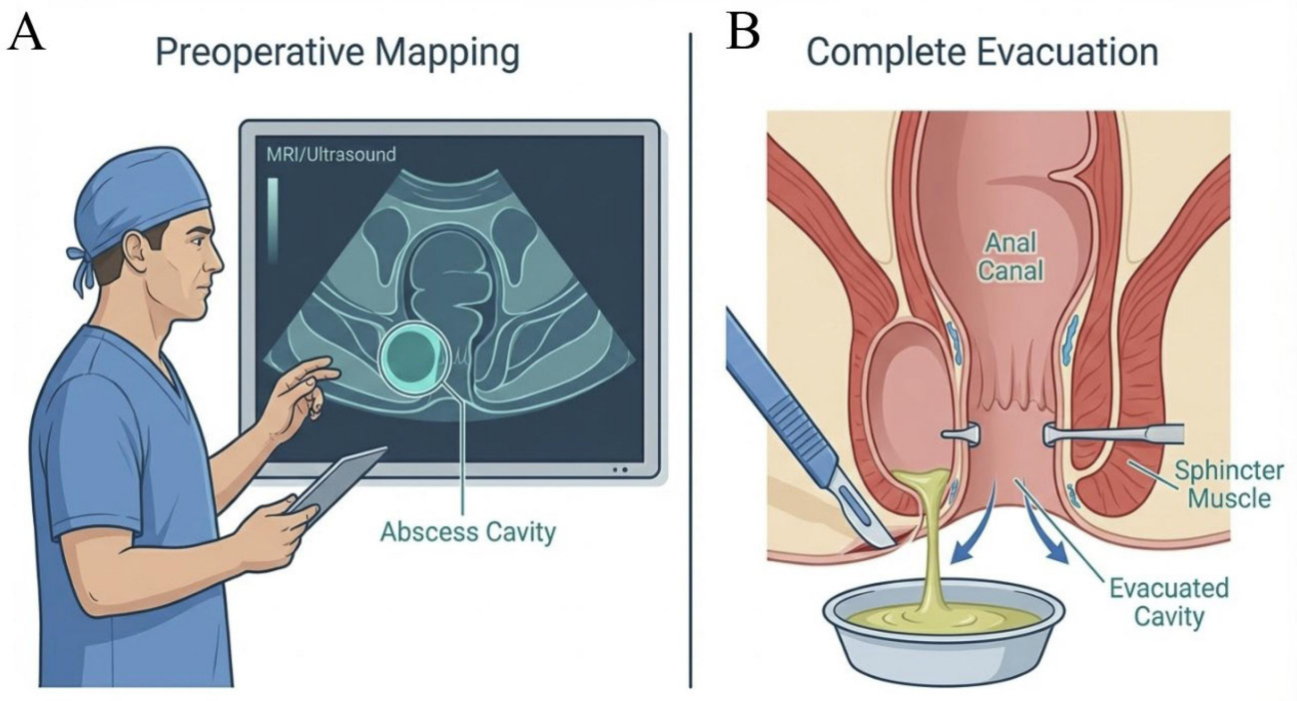
Optimization of incision and drainage (I&D) in pediatric perianal abscesses. **(A)** Preoperative mapping using magnetic resonance imaging (MRI) or ultrasound enables precise delineation of abscess boundaries and their spatial relationship to the anal sphincter complex. **(B)** Surgical execution prioritizes complete evacuation of the abscess cavity through a minimal incision, while strictly preserving the integrity of the internal and external anal sphincter muscles. This anatomy-guided approach minimizes iatrogenic sphincter injury during the acute inflammatory phase.

In older children (>12 months), perianal abscesses are more frequently associated with true fistulizing disease (Figure 2). Consequently, incision and drainage (I&D) in this cohort warrants a meticulous examination under anesthesia (EUA) to identify complex pathology. Recent clinical evidence underscores that the primary determinants of I&D success are the thorough evacuation of the pus cavity and the integration of preoperative imaging (ultrasound/MRI). By accurately navigating complex anatomical tracks and eliminating intraoperative residue, these measures significantly reduce the incidence of secondary fistula formation and long-term recurrence (24).

The success of I&D extends beyond the operative procedure, requiring a multimodal perioperative strategy to ensure favorable outcomes. Multimodal pain management, integrating both pharmacologic and non-pharmacologic interventions, is essential for enhancing patient cooperation and accelerating recovery. Postoperative wound care, centered on daily cleansing and meticulous hygiene, remains a cornerstone of infection prevention; however, its efficacy relies heavily on parental education and compliance. While adjunctive antibiotic therapy is frequently employed, its use should be judicious-guided by systemic symptoms, the extent of cellulitis, and microbial culture results-to mitigate the risk of antimicrobial resistance. Furthermore, structured long-term follow-up is imperative for the early detection of recurrence or the identification of occult fistulas, which remain the primary drivers of treatment failure.

### 4.2 Management of concomitant fistula-in-ano (FIA)

The identification of a fistula tract during the acute phase presents a surgical dilemma. The choice between primary fistulotomy and seton placement is dictated by the amount of sphincter muscle involved:

Primary Fistulotomy (Lay-open): This is reserved exclusively for low-transsphincteric or intersphincteric fistulas where the tract is superficial. While primary fistulotomy (incision–drainage plus fistulotomy, IDF) yields lower recurrence rates compared to I&D alone, it carries a non-negligible risk of minor incontinence. Therefore, it is contraindicated in cases where the fistulous tract traverses a significant portion of the external anal sphincter.

**Figure 3:**
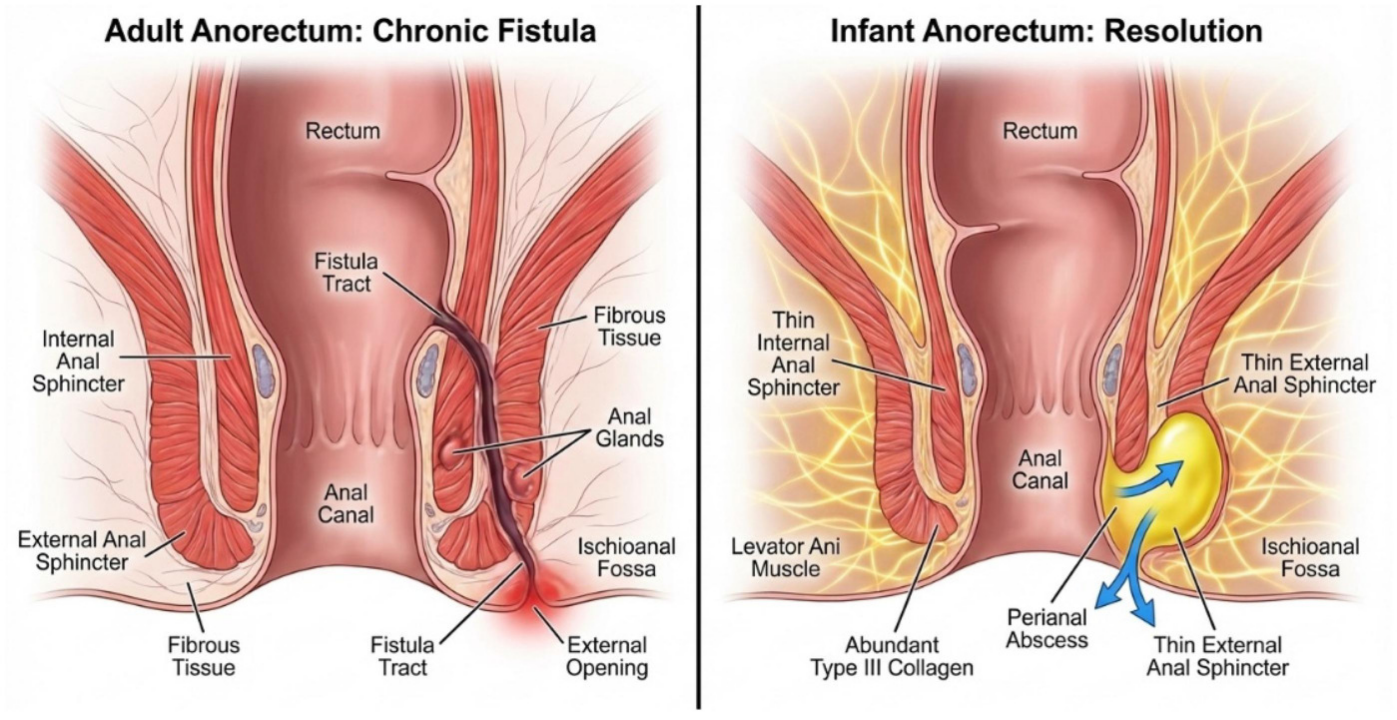
Perianal abscess outcomes in adults and infants. Adults: rigid anatomy, reduced elasticity, and fibrotic repair favor abscess persistence and chronic fistula formation. Infants: High tissue plasticity, elasticity, and type III collagen enrichment promote spontaneous resolution and limit fistula development.

Seton Placement (Drainage Seton): For high-transsphincteric fistulas or when local inflammation obscures anatomy, placing a non-cutting seton (e.g., vessel loop or suture) is the preferred “Bridge-to-Surgery” strategy. The seton maintains patent drainage, prevents premature skin closure, and facilitates the maturation of the fistula tract, allowing for a safer definitive repair once inflammation subsides. This approach minimizes the risk of acute sphincter injury during the fragile inflammatory state (Figure 3).

### 4.3 Emerging minimally invasive techniques: from energy modalities to bio-interactive materials

Recent advancements in pediatric coloproctology have catalyzed a shift toward ultra-minimally invasive interventions that prioritize the functional preservation of the anal sphincter. Traditionally adult-centric techniques, such as Video-Assisted Anal Fistula Treatment (VAAFT) (25) and Fistula-tract Laser Closure (FiLaC) (26), are increasingly adapted for the pediatric population. These modalities facilitate the precise identification and thermal ablation of the fistula tract under direct visualization, significantly reducing postoperative pain and the risk of fecal incontinence. However, despite their theoretical advantages, these energy-based treatments are currently reserved for complex or refractory cases in specialized tertiary centers, pending the availability of robust long-term efficacy and safety data in children.

Beyond these mechanical and thermal interventions, the frontier of anal fistula therapy has expanded into the realm of immunomodulatory bioengineering. A landmark development in this trajectory is the design of a quaternized molecular brush-grafted injectable microgel (GAA@CNT-g-PVBTMA), which marks a transition from passive drainage to active biochemical modulation. This microgel employs a unique porous, fragmented morphology that not only provides a scaffold for effective fistula filling but also maintains essential exudate drainage. Most notably, the material leverages electrostatic interactions via its quaternized molecular brushes to actively scavenge pro-inflammatory cytokines, achieving clearance rates of 92.6% for TNF-α and 92.5% for IL-1β. Experimental evidence indicates that this targeted suppression of the local inflammatory microenvironment significantly accelerates epithelialization and tissue regeneration. By integrating mechanical support with potent anti-inflammatory properties, such bio-interactive materials offer a promising non-surgical alternative that aligns with the robust regenerative potential of the pediatric population while circumventing the risks of iatrogenic sphincter trauma (27).

Management of pediatric perianal absences has evolved into a risk-stratified framework centered on sphincter preservation. While incision and drainage (I&D) remain the acute-phase standard, definitive fistula repair is strictly dictated by anatomical height: primary fistulotomy is reserved for superficial tracts, whereas non-cutting setons serve as the bridge for high-type cases. The transition from passive mechanical debridement to active biological modulation integrates energy modalities such as VAAFT and FiLaC with immunomodulatory biomaterials. This evolution is conceptually aligned with the pediatric “Developmental Healing Axis”, aiming to optimize fistula closure while minimizing iatrogenic functional risks.

## 5 Conservative management: leveraging the developmental healing window

### 5.1 Clinical rationale: “Wait-and-See”

For pediatric perianal absences, particularly in infants under 12 months, the therapeutic philosophy has shifted from aggressive intervention to a “wait-and-see” approach. Unlike the adult pathophysiology driven by chronic cryptoglandular infection, infantile abscesses are often self-limiting, driven by transient androgen surges and immature sebaceous glands. Consequently, conservative management serves as the first-line strategy, aiming to maximize the potential for spontaneous resolution while avoiding the sequelae of radical surgery-such as anal incontinence, scar deformity, and recurrence due to iatrogenic trauma to underdeveloped tissues (28–30). Current consensus advocates a stepwise protocol: initiating with non-operative hygiene and antibiotic measures, reserving invasive procedures solely for non-responsive, complex, or systemic cases (31, 32).

### 5.2 Biological basis: regenerative advantage

The apparent efficacy of conservative management in infants may not be solely empirical but could partially reflect distinct biological characteristics of pediatric perianal tissues. Available evidence suggests that neonatal and early-life tissues tend to exhibit a higher proportion of Type III collagen, a component of the extracellular matrix that has been associated with more regenerative and less fibrotic healing responses (33, 34). In addition, developmental studies indicate that local progenitor cell activity and tissue remodeling capacity may be relatively enhanced during early life stages (35).

While these biological features may partially account for the higher rates of spontaneous resolution observed in infants, the available evidence is predominantly indirect and does not establish a definitive causal relationship. Accordingly, these findings are better regarded as providing supportive mechanistic context rather than conclusive proof.

Against this background, the proposed “Developmental Healing Axis” should be interpreted as a conceptual framework intended to integrate age-related differences in tissue repair capacity and to contextualize variability in clinical outcomes. It is not presented as a validated biological pathway or as a basis for direct clinical decision-making, but rather as a hypothesis-generating model that may inform future research and refinement of treatment strategies.

### 5.3 Evidence-based conservative protocols

Effective conservative management comprises a trial of infection control, local care, and systemic support.

Judicious Antibiotic Therapy: For early-stage, non-fluctuant abscesses with surrounding cellulitis, systemic broad-spectrum antibiotics are employed to suppress bacterial load and prevent progression. The duration and choice of agents should be guided by local resistance patterns to minimize microbiome disruption (36–38).

Hygiene and Hydrotherapy: Integrating warm sitz baths (2–3 times daily) serves a dual mechanism: improving local microcirculation to accelerate resolution and facilitating the spontaneous drainage of purulent material. Strict perianal hygiene acts as a barrier against secondary colonization by fecal flora.

Systemic Exclusion: While treating the local pathology, it is imperative to rule out underlying systemic etiologies. Persistent or recurrent abscesses in older children warrant screening for Crohn's disease, immunodeficiency (e.g., chronic granulomatous disease), or anatomical malformations (39, 40).

### 5.4 Criteria for escalation

Conservative management may offer a favorable balance between efficacy and safety for the infant population, aligning with their observed physiological regenerative potential. However, this approach requires rigorous monitoring. The transition from conservative care to surgical intervention (Section 3) is indicated by failure of spontaneous resolution within 7–10 days, expansion of the abscess, or development of systemic toxicity. By strictly adhering to this biological rationale, clinicians can maximize functional preservation while ensuring timely control of the infectious source (Table 2).

**Table 2:** Quantified clinical outcomes: CM vs. ID vs. IDF.

Treatment	Core strategy	Age range	Cure (%)	Recurrence (%)	Key advantage	Key limitation
CM	Observation/Hygiene	<12 m	80–95	5–15	Non-invasive; preserves tissue	Slow resolution
ID	Simple Drainage	Any age	80–85	10–20	Immediate symptomatic relief	High recurrence risk
IDF	Drainage + Fistulotomy	>6 m	95–98	2–5	Definitive cure; lowest recurrence	Invasive; requires anesthesia

### 5.5 Conservative management in pediatric perianal abscesses

Conservative management (CM) has been increasingly recognized as a feasible initial approach for selected infants with perianal abscesses, particularly in cases with mild and localized disease (41). In clinical practice, this strategy is most considered in otherwise stable infants (typically younger than 12 months of age) presenting with non-fluctuant lesions and without systemic signs of infection, such as fever or leukocytosis (42).

CM generally focuses on supportive local care rather than immediate surgical intervention. Commonly reported measures include regular warm sitz baths or gentle cleansing several times daily, often combined with the use of barrier creams to protect perianal skin integrity. The role of systemic antibiotics remains controversial, but they are typically reserved for cases with progressive erythema or suspected secondary infection.

Close clinical monitoring is a key component of this approach. Early reassessment-usually within 24–48 h-is recommended to evaluate disease progression. In patients with stable or improving findings, continued observation for up to 1–2 weeks may allow for spontaneous resolution, which has been reported in a proportion of infant cases.

Importantly, CM should not be regarded as a fixed pathway but rather as a dynamic, response-guided strategy (43). Escalation to surgical intervention is generally considered when clinical deterioration occurs, including the development of systemic symptoms, rapid enlargement or fluctuation of the lesion, or failure to improve over a short observational period.

Taking together, current evidence suggests that CM may offer a reasonable balance between safety and efficacy in carefully selected infants, although variability across studies highlights the need for individualized decision-making and further prospective validation.

## 6 Conclusions and future perspectives

This review systematically elucidates that the management of pediatric perianal abscesses must transition from a conventional “one-size-fits-all” surgical approach to an age-stratified, biologically informed paradigm. Our synthesis of anatomical and molecular evidence, particularly the age-dependent enrichment of Type III collagen and distinct cell adhesion protein profiles in neonatal models, provides a potential mechanistic basis for the superior self-healing capacity observed in infants. These findings suggest the feasibility of a conservative-first strategy for younger children, which is conceptually aligned with the proposed “Developmental Healing Axis”. This approach seeks to promote spontaneous resolution while minimizing iatrogenic risks associated with radical fistula excision. Conversely, for older children, clinical decision-making should remain individualized, balancing surgical precision with the evolving chronicity of the disease to ensure optimal functional preservation. We acknowledge that the clinical observations integrated into this review are characterized by significant heterogeneity in terms of patient populations, diagnostic definitions, and follow-up durations across studies. Consequently, while the proposed strategies align with current evidence, they should be interpreted as contextual recommendations rather than universal clinical mandates.

Moving forward, the frontier of pediatric coloproctology lies in the synergy between precision medicine and bio-interactive engineering. Future research should pivot toward identifying molecular biomarkers through multi-omics landscapes and developing “smart” immunomodulatory biomaterials, such as cytokine-scavenging microgels, to actively modulate the inflammatory microenvironment. Furthermore, exploring the role of the perianal microbiome and the long-term translational efficacy of minimally invasive energy modalities (e.g., VAAFT/FiLaC) remains essential. Ultimately, by bridging basic science insights with interdisciplinary clinical practice and enhanced caregiver education, we can refine therapeutic outcomes and safeguard the long-term quality of life for the pediatric population.
